# Combination therapy of cold atmospheric plasma (CAP) with temozolomide in the treatment of U87MG glioblastoma cells

**DOI:** 10.1038/s41598-020-73457-7

**Published:** 2020-10-05

**Authors:** Eda Gjika, Sonali Pal-Ghosh, Megan E. Kirschner, Li Lin, Jonathan H. Sherman, Mary Ann Stepp, Michael Keidar

**Affiliations:** 1 Department of Mechanical and Aerospace Engineering, School of Engineering and Applied Science, The George Washington University, Washington, DC USA; 2 Department of Anatomy and Cell Biology, The George Washington University School of Medicine & Health Sciences, Washington, DC USA; 3 Department of Neurological Surgery, The George Washington University School of Medicine & Health Sciences, Washington, DC USA

**Keywords:** Plasma physics, Cancer therapy

## Abstract

Cold atmospheric plasma (CAP) technology, a relatively novel technique mainly investigated as a stand-alone cancer treatment method in vivo and in vitro, is being proposed for application in conjunction with chemotherapy. In this study, we explore whether CAP, an ionized gas produced in laboratory settings and that operates at near room temperature, can enhance Temozolomide (TMZ) cytotoxicity on a glioblastoma cell line (U87MG). Temozolomide is the first line of treatment for glioblastoma, one of the most aggressive brain tumors that remains incurable despite advancements with treatment modalities. The cellular response to a single CAP treatment followed by three treatments with TMZ was monitored with a cell viability assay. According to the cell viability results, CAP treatment successfully augmented the effect of a cytotoxic TMZ dose (50 μM) and further restored the effect of a non-cytotoxic TMZ dose (10 μM). Application of CAP in conjunction TMZ increased DNA damage measured by the phosphorylation of H2AX and induced G2/M cell cycle arrest. These findings were supported by additional data indicating reduced cell migration and increased αvβ3 and αvβ5 cell surface integrin expression as a result of combined CAP–TMZ treatment. The data presented in this study serve as evidence that CAP technology can be a suitable candidate for combination therapy with existing chemotherapeutic drugs. CAP can also be investigated in future studies for sensitizing glioblastoma cells to TMZ and other drugs available in the market.

## Introduction

Glioblastoma multiforme (GBM) is the most common and aggressive type of malignant brain tumor among adults. The current standard of care for GBM includes maximum debulking surgery, radiation therapy and treatment with the alkylating agent temozolomide (TMZ) also referred to as Temodar. Although these therapies aim to prolong survival and improve quality of life, their long-term efficacy is arguable as the median survival rate among patients continues to remain in the range of 9–12 months. The incurability of GBM has been attributed to its profound therapy resistance^1–4^. The need for improving survival outcomes has created a high demand for novel treatment methods that can enhance or restore the cytotoxicity of TMZ. The cold atmospheric plasma (CAP) technology is being proposed for application in combination therapy to enhance the effect of TMZ. CAP can be artificially produced in laboratory settings by applying a high alternating electric current through a gas which results in a plasma glow discharge. This type of plasma generation only thermalizes the electrons and not the heavier ions and neutral species, therefore the overall plasma system remains near room temperature, making it ideal for biomedical applications as it operates under the threshold of thermal damage. Although the mechanism of action of CAP is still under investigation, it is suggested that CAP operates by inducing physical effects via CAP-produced ultraviolet rays, heat and electromagnetic fields, and chemical effects via CAP-produced reactive oxygen and nitrogen species (RONS)^5,6^.

The studies performed with CAP as a standalone treatment modality serve as evidence that this type of technology can be a suitable candidate for combination therapy. In the last decade, scientists and engineers investigated CAP in vivo and in vitro for the treatment of several types of cancers, including glioblastoma. The studies reported a reduction in cancer cell proliferation and an increase in apoptosis in vitro*,* and a reduction in tumor volume in vivo as a result of CAP treatment^7–12^. In 2018, the third clinical trial ever performed with CAP presented the application of this technology for the treatment of head and neck cancer. According to the investigators, CAP therapy resulted in a reduction of wound odor, decreased demand for pain medication, improvement in social function and a positive emotional effect in most patient cases investigated^13^.

Studies have revealed that an effective CAP-only and TMZ-only treatment can result in single and double-strand DNA breaks that induce cell cycle arrest at G2/M and eventually lead to apoptosis^4,14,15^. Additionally, administration of TMZ has been reported to exert an effect in the expression levels of cell surface receptors, known as integrins, that are responsible for cancer cell migration, survival and angiogenesis^16^. αvβ3 and αvβ5 are two of the main integrins positively associated with cell migration and drug resistance in glioblastoma^17,18^. However, to our knowledge they have never been evaluated with CAP therapy. Thus far, cell migration, integrins and CAP have only been discussed in the context of wound healing^19–21^. Therefore, investigating the molecular anticancer mechanisms in combined CAP–TMZ treatment is significant in improving our understanding of the role of CAP therapy.

In this investigation we evaluated the effect of CAP applied in conjunction with TMZ on the treatment of U87MG glioblastoma cells. We assessed whether CAP could enhance the effect of a cytotoxic TMZ dose, and described treatment outcomes in terms of cell viability, cell cycle distribution, DNA damage, cell migration and αv, αvβ3, and αvβ5 cell surface integrin expression.

## Results

### Combined CAP–TMZ treatment causes a reduction in cell viability compared to TMZ

**Figure 1:**
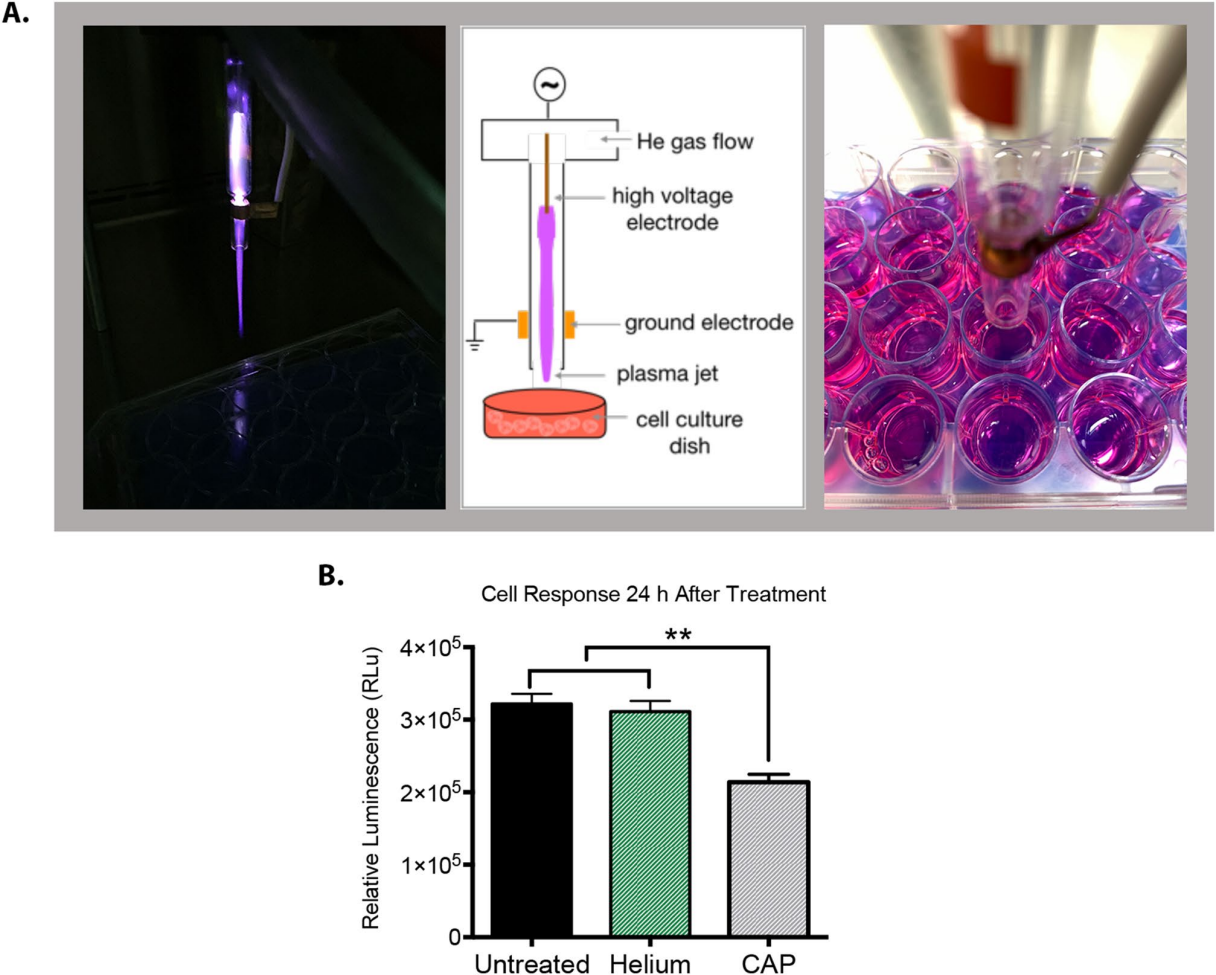
Cell response to helium and CAP jet. (**A**) The actual plasma jet, a schematic explanation of the generation of the plasma jet and the size of the jet nozzle in relation to the wells treated with plasma. (**B**) Glioblastoma cell response to 180 s-CAP and 180 s-helium at 24 h after treatment. Cell response can be attributed to CAP because helium does not create any lasting effects. The asterisk indicates statistical significance compared to the untreated condition unless otherwise noted.

The anti-tumor effect of CAP as a standalone therapy in the treatment of glioblastoma and other cancers has been previously investigated^22–30^. Positive treatment outcomes reported by several studies make CAP a promising treatment strategy to be evaluated for enhancing sensitivity to TMZ in glioblastoma. CellTiter-Glo 2.0 luminesce assay was utilized for monitoring cell response. The luminescent signal of this assay corresponds to cell metabolic activity, which serves as a proxy for cell viability. The CAP jet utilized in this study (Fig. 1A) was generated via ionization of helium gas. Exposure to helium for an extended period of time can lead to evaporation of liquid and precipitation of salts by turning the media into a hypertonic solution. To eliminate any potential effects on the cells due to helium, the media was replaced after CAP treatment. We investigated cell response to treatment with 180 s-helium at 24 h after treatment. According to Fig. 1B, the selected helium treatment parameters had no effect on cell viability, confirming that the decrease in cell viability was entirely attributed to CAP treatment.

**Figure 2:**
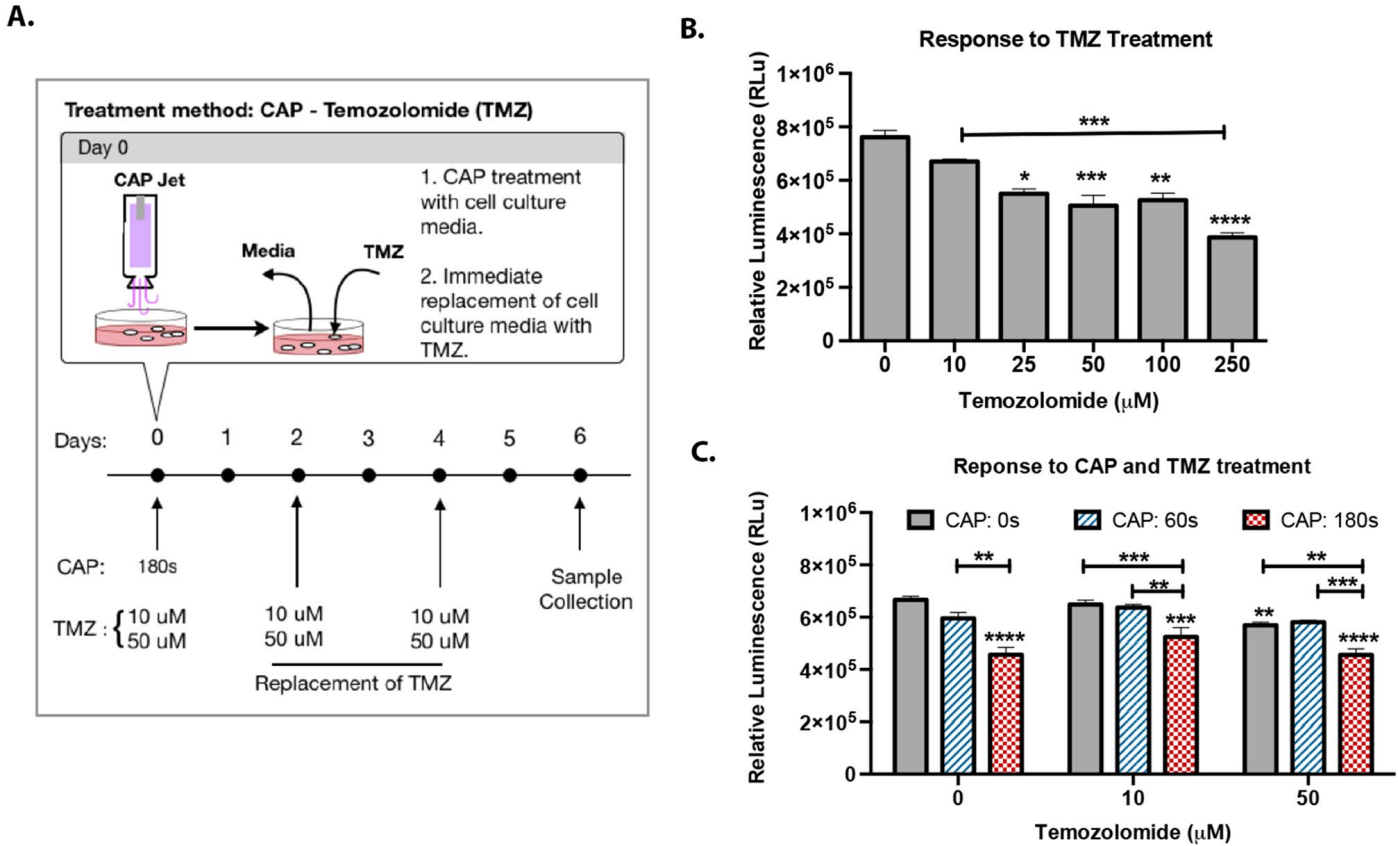
Inhibition of cell proliferation by temozolomide. (**A**) U87MG glioblastoma cells were treated with 10 μM, 25 μM, 50 μM, 100 μM and 250 μM of temozolomide for 6 days with the drug being replaced on alternate days. Cell response was recorded 6 days after plasma treatment. (**B**) Schematic representation of CAP and TMZ treatment over the course of 6 days. (**C**) Treatments with 180 s-CAP and 10 μM or 50 μM TMZ enhances cell response to drug treatment in comparison to TMZ alone in a dose-dependent manner, providing maximal outcomes with the drug being replaced every alternate day for 6 days. The luminescent signal measured with CellTiter-Glo 2.0 assay, is proportional to the number of viable cells. Error bars indicate the standard error of the mean, and the asterisks indicate statistical significance compared to the untreated condition unless otherwise noted.

Cell response to TMZ treatments of 10, 25, 50, 100 and 250 μM was evaluated to determine suitable TMZ doses. The U87MG cell response to different TMZ doses was monitored over the course of 6 days with the drug treatment administered on alternate days (Day 0, 2 and 4). Figure 2A details the protocol followed for CAP and TMZ treatment. TMZ displayed no significant cytotoxic effect within the first 24 h of treatment (data not shown). Figure 2B reveals the cell response to TMZ treatment 6 days after the application of the initial drug dose (Day 0). On day 6, the impact of TMZ on cell response was more pronounced with drug doses of 25–250 μM causing a significant dose-dependent inhibition in cell growth. An IC_50_ of 25.8 μM was calculated using Prism GraphPad (data not shown). Based on these findings, we selected drug doses within the range present in blood plasma and cerebrospinal fluid (CSF) of treated patients. According to a previous study, TMZ administered orally at 150–200 mg/m^2^ cm (approved dose) results in a peak blood plasma concentration of 3–15 µg/ml corresponding to 15–77 µM drug dose^31^. Whereas a single daily treatment of TMZ at 75 mg/m^2^ was reported to be equivalent to a mean peak concentration of 10–25 µM in CSF^31–33^. Therefore, in our study we investigated cell viability response to CAP treatment in the presence of 50 μM (cytotoxic) and 10 μM (non-cytotoxic) drug dose on day 6 (Fig. 2C). Cell viability results from combined CAP–TMZ treatment were compared with untreated (control), CAP and TMZ treated conditions. According to Fig. 2C, a single CAP treatment of 60 s was ineffective in improving cell response to the 10 μM non-cytotoxic TMZ dose and did not enhance the effect of the 50 μM TMZ cytotoxic dose. In contrast, the 180 s-CAP treatment successfully improved the effect of both TMZ doses (Fig. 2C). Results for the combination therapy with 180 s-CAP − 50 μM TMZ revealed a statistically significant reduction in cell viability of 20% compared to the drug. The data indicated that plasma should cause at least a 30% reduction in viability as a standalone therapy in order to create an irreversible cell death effect when administered with TMZ. Therefore, the CAP device parameters should be selected accordingly.

### Accumulation of cells in the G2/M phase of the cell cycle

**Figure 3:**
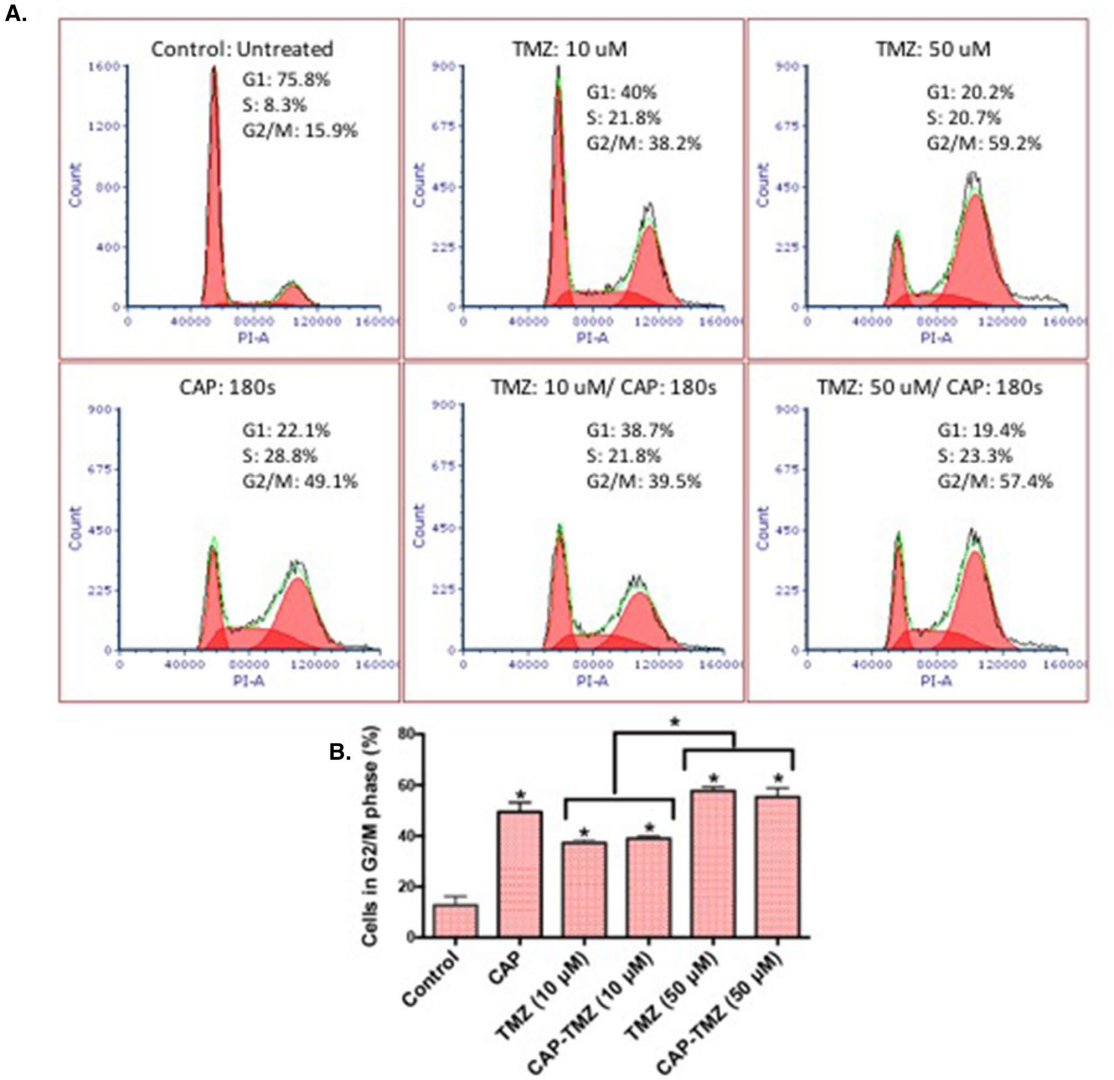
Cell cycle arrest in G2/M phase. (**A**) Cell distribution in each phase of the cell cycle for control, CAP, TMZ and CAP–TMZ variables. (**B**) Statistical significance in the G2/M phase for U87MG cells 6 days after treatment with 180 s of plasma, 10 and 50 μM temozolomide and CAP–TMZ combined. The drug was replaced every alternate day. The results were reproduced with a set of six samples per condition. Error bars indicate the standard error of the mean, and the asterisk indicates statistical significance compared to the untreated condition unless otherwise noted.

The progression of the cells through the cell cycle was investigated to acquire a better understanding of the cell viability results. Flow cytometry was used to assess the number of cells in the three distinct phases of the cell cycle (G1, S, and G2/M). Cells that undergo replication progress from G1 phase into the S phase for DNA synthesis followed by the G2 phase, where they prepare for duplication in the M phase. Figure 3A shows the percent cell distribution in each phase of the cell cycle for control (untreated), CAP, TMZ and combined CAP–TMZ treated cells. The cells were analyzed after 6 days of treatment. According to Fig. 3B, treatment of the U87MG glioblastoma cells with CAP for 180 s, can lead to a four time higher percentage of cells in the G2/M phase of the cell cycle compared to control. TMZ treatment also induced a delay in the progression of cells through the cell cycle in a TMZ dose dependent manner (10–50 μM). Interestingly, TMZ and combined CAP–TMZ conditions revealed a similar cell accumulation in G2/M making it unclear whether CAP had a role in improving cell response to TMZ treatment. Therefore, an additional investigation was necessary for exploring the role of CAP in combined CAP-TMZ therapy.

### DNA damage as a result of combined CAP–TMZ treatment

**Figure 4:**
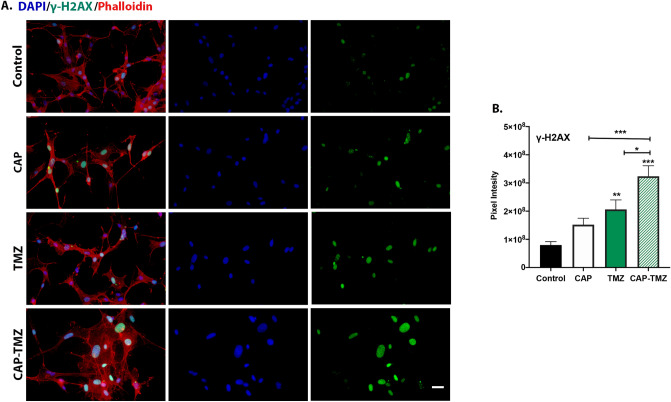
Influence of CAP on TMZ induced DNA damage. (**A**) The immunofluorescence images of glioblastoma U87MG cells antibodies against γ-H2AX for the following variables: untreated control, 180 s CAP (1 treatment), 50 μM TMZ (3 treatments) and combined CAP–TMZ treatments on day 6. (**B**) The data represents the level of phosphorylated H2AX in each treatment group and is expressed as the mean pixel intensity per cell. These values are calculated by dividing the sum intensity of each image by the total number of cells in the image. A total of 24 fields were analyzed per condition. Error bars indicate the standard error of mean, and the asterisk indicates statistical significance to untreated control conditions unless otherwise noted. Scale bar: 14 μm.

Cancer cells respond to DNA damage with cell cycle arrest and entry into apoptosis or other cell death mechanisms. Based on our previous cell viability findings, we hypothesized that CAP treatment induces a greater degree of DNA damage in TMZ treated glioma cells. To test this hypothesis, we assessed the extent of DNA damage in the cells by studying the expression of γ-H2AX on day 6 after treatment. γ-H2AX, a histone family member generated by the phosphorylation of H2AX at Serine 139, is considered as the most sensitive marker for evaluating DNA double-strand breaks (DSBs). γ-H2AX is expressed at very low-level during cell cycle progression. When DNA double-stranded breaks (DSBs) are detected, cells upregulate γ-H2AX^34^. Figure 4A shows representative images of cells stained with γ-H2AX clearly exhibiting a steady increase in γ-H2AX expression levels following treatment with CAP, TMZ and combined CAP–TMZ. Quantification of data from 24 fields for each variable are shown in Fig. 4B. The mean pixel intensity per cell was obtained by dividing the sum pixel intensity of each field by the total number of cells as indicated by DAPI—a nuclei stain. Figure 4B highlights the efficacy of CAP in combined CAP–TMZ treatment, by revealing that CAP–TMZ is responsible for inducing a 30% increase in γ-H2AX expression compared to TMZ treated cells. Additionally, TMZ and CAP treatments alone induced a 1–2-fold increase in the expression levels of γ-H2AX compared to the control condition. Although not statistically significant, TMZ also showed slightly higher levels of γ-H2AX than CAP. Similarly to the cell viability results, these findings confirmed the role of CAP in enhancing cell response to TMZ treatment.

### Glioblastoma cells reduce their migration in response to combined CAP–TMZ treatment

**Figure 5:**
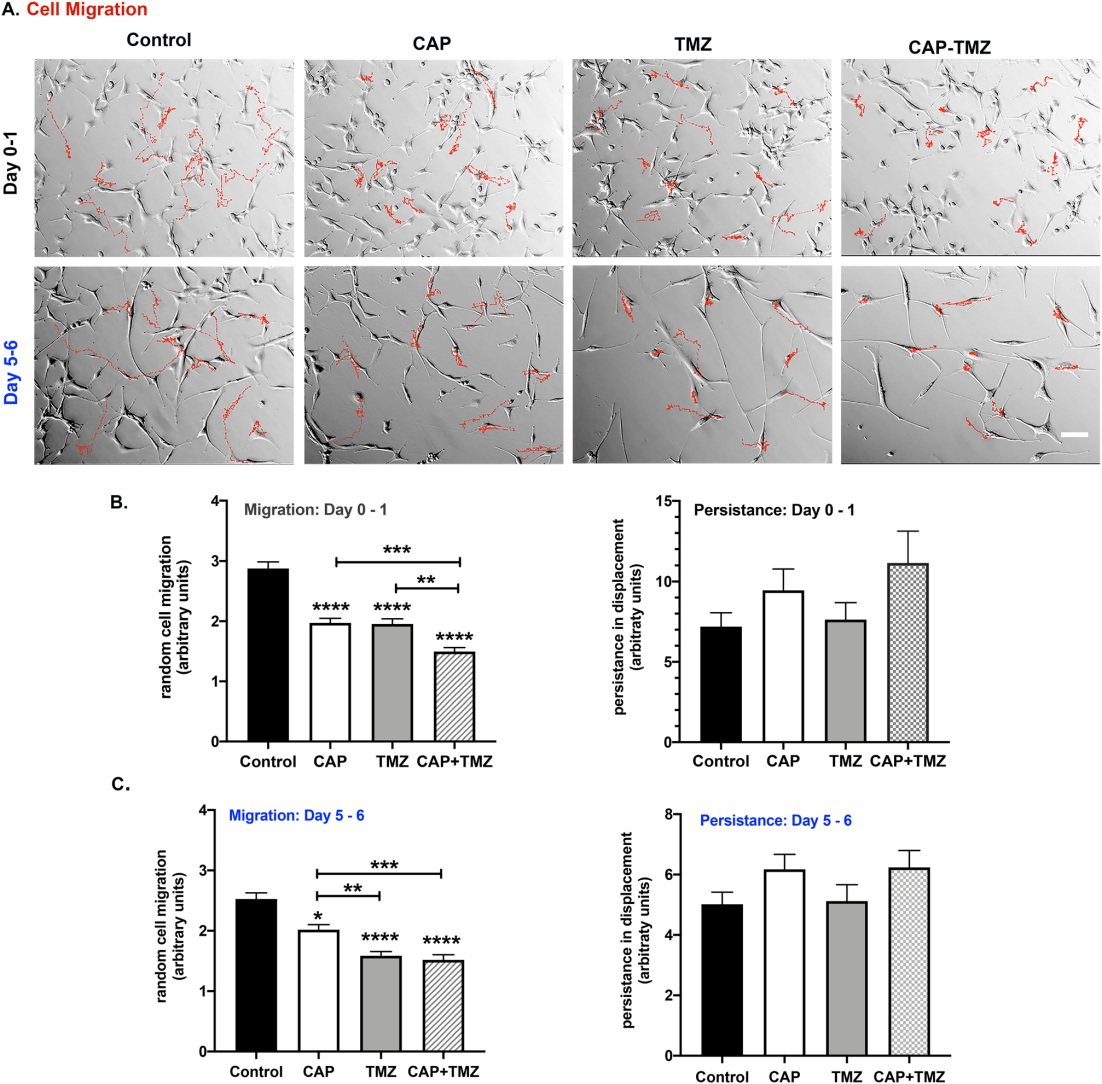
CAP reduces the rate of cell migration in U87MG cells. (**A**) Control (untreated), CAP-only (180 s), TMZ-only (50 μM) and combined CAP–TMZ treated cells were tracked using a time-lapse microscopy. Tracks of ten representative cells studied over the 16-h time period are indicated in red for Day 0–1 and Day 5–6. Images were obtained every 10 min until 100 images were acquired (16 h, 40 min). The tracks traveled by cells in the CAP–TMZ condition are shorter than those for the control variables (**B** and **C**). The graphs indicate the rate of cell velocity and cell displacement of 60 cells per variable. Persistence was measured as the ratio between net and total displacements. Error bars indicate the standard error of the mean, and the asterisk indicates statistical significance to untreated control conditions unless otherwise noted. Scale bar: 14 μm.

Cell migration and invasion are crucial steps implicated in many physiological events including tumor treatment and suppression^35^. A previous investigation showed that CAP can significantly reduce epithelial cell migration^19^ while the effect of combined CAP–TMZ treatment on cell migration is yet to be investigated. Using a time-lapse microscopy, we assessed the rate of cell migration of U87MG glioblastoma cells treated with 180 s of plasma (single treatment) and repeated treatments with 50 μM TMZ. The trend in cell migration in untreated (control), TMZ-only, CAP-only and combined CAP–TMZ treated cells was monitored for the initial 16 h after treatment (Day 0–1) and in the last 16 h (Day 5–6) before the treatment was completed. Representative tracks obtained from ten cells that did not undergo mitosis are shown in Fig. 5A. The red tracks indicate the paths taken by the cells during the time in which 100 images were taken. The tracks from the combined CAP–TMZ treated cells are shorter than those from untreated, CAP-only and TMZ-only treated cells for both time points investigated. Quantification of data from 60 cells tracked for each variable are shown in Fig. 5B,C. The effect of CAP on cell migration can be observed within the first 16 h of treatment. CAP appeared to significantly reduce cell migration in TMZ treated cells. As shown in Fig. 5, cells exposed to a combined treatment of CAP–TMZ migrated significantly slower than those exposed to CAP or TMZ treatments (days 0–1). The decrease in the rate of cell migration is about 30–40%. By contrast, when migration rates are assessed several days after treatment (days 5–6), cells exposed to combined CAP–TMZ treatment migrate at the same rate as the TMZ treated cells but, have a significantly slower rate of migration than control and CAP treated cells. The cells in combined CAP–TMZ and TMZ treatment conditions also appear more spread and elongated compared to the ones in the CAP and control panel (Fig. 5A). Persistence, which represents the directionality of the cell’s motion, was evaluated for all variables. Persistence was measured as the ratio between net and total cell displacements (Fig. 5B,C). Cells treated with CAP–TMZ therapy show changes in persistence compared to the control groups. These results are significant because cell migration plays a crucial role in chemo resistance and invasion.

### Combined CAP–TMZ treatment increases expression of αv, αvβ3 and αvβ5 cell surface integrins

Integrins are polarized integral membrane glycoproteins that are present at the cell surface in both inactive and active conformations. Their extracellular domains bind to extracellular matrix molecules outside cells inducing conformational changes transmitted to their intracellular signaling domains via changes in the conformation of the amino acids that make up their transmembrane domains. Integrin engagement extracellularly transduces signals to the nucleus intracellularly. As a result, integrin family members integrate intracellular signaling events that control adhesion, migration, cell survival, proliferation, and differentiation. Changes in integrin surface expression can lead to changes in cell migration rates. Integrins are heterodimeric transmembrane receptors consisting of one α and one β chain; among the αv class integrins, αvβ3 and αvβ5 have been targeted in cancer treatment and have been reported to play prominent roles in glioma cell adhesion, migration, angiogenesis and therapy resistance^17,36–42^. Moreover these integrins are considered as suitable therapeutic targets for malignant gliomas and have also been investigated in U87MG glioblastoma cells^18^.

**Figure 6:**
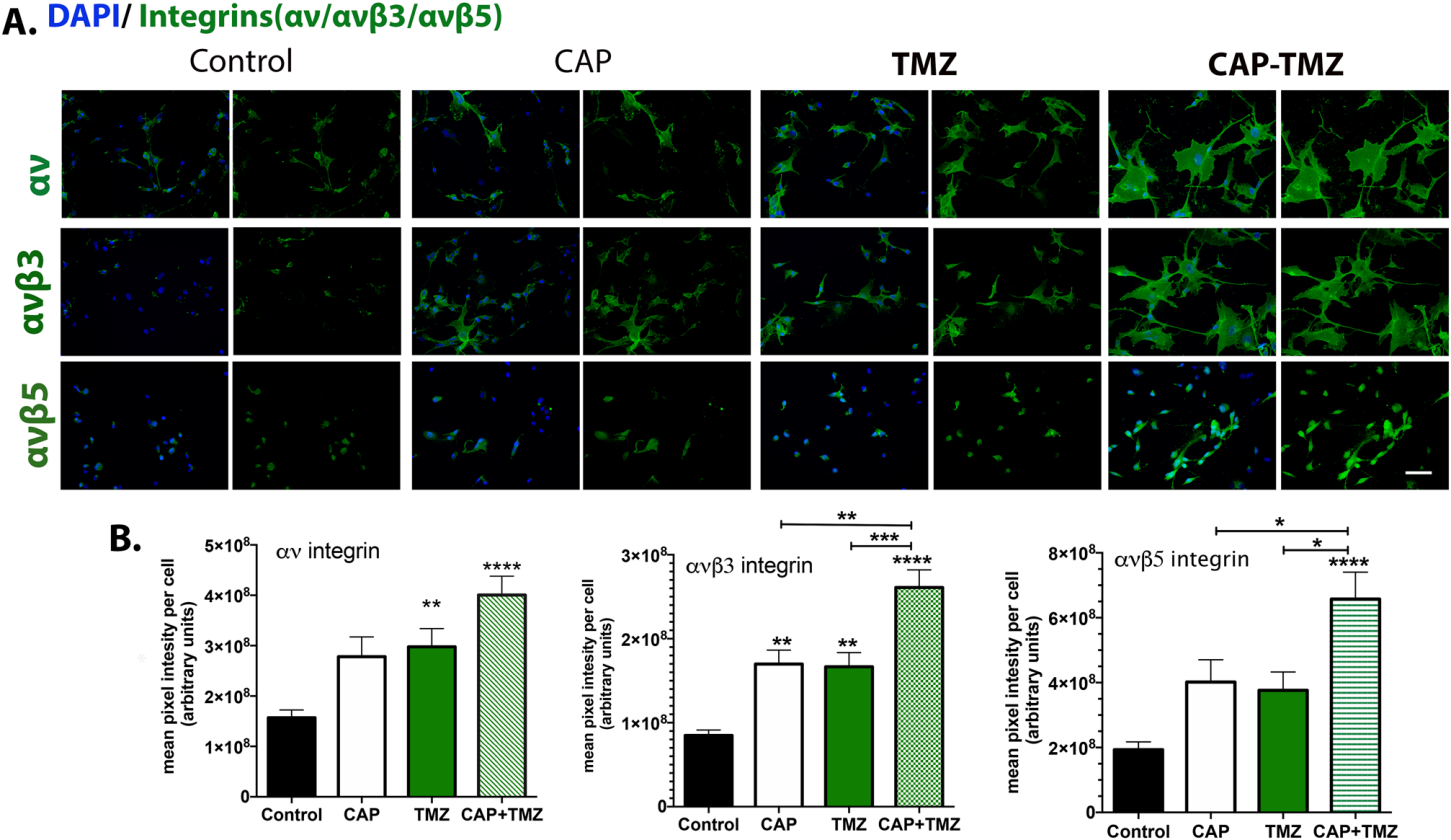
Cell surface integrin expression levels of total αv, αvβ3 and αvβ5. (**A**) The immunofluorescence images of glioblastoma U87MG cells are shown for the following variables: untreated control, 180 s CAP (1 treatment), 50 μM TMZ (3 treatments) and combined CAP–TMZ treatments on day 6. Images of cells expressing total αv, αvβ3 and αvβ5 integrins are shown in green and the nuclei in blue. (**B**) The data represents the level of integrin in each treatment group and is expressed as the mean pixel intensity per cell. These values are calculated by dividing the sum intensity of each field by the total number of cells in the field. A total of 12 fields were obtained and analyzed per condition. Error bars indicate the standard error of mean, and the asterisk indicates statistical significance to untreated control conditions unless otherwise noted. Combined CAP–TMZ treated cells express more αv, αvβ3 and αvβ5 integrins than control, CAP or TMZ treated cells. Error bars indicate the standard error of the mean, and the asterisk indicates statistical significance to untreated control conditions unless otherwise noted. Scale bar: 50 μm.

To determine if expression levels of αv, αvβ3 and αvβ5 cell surface integrins were impacted by combined CAP–TMZ treatment, glioblastoma cells were fixed on day 6 of treatment and used for immunofluorescence studies without permeabilization. Untreated cells (control), cells treated with CAP, TMZ and combined CAP–TMZ were stained with antibodies against total αv, αvβ3 and αvβ5 integrins. The data presented in Fig. 6A and quantified in Fig. 6B revel significantly higher levels of total αv, αvβ3 and αvβ5 cell surface integrins present in combined CAP–TMZ treated cells compared to control. Additionally, combined CAP–TMZ treated cells consistently expressed more αv, αvβ3 and αvβ5 cell surface integrins than CAP or TMZ treated cells; even when statistical significance between the three types of treatment was not observed (as in the case of αv integrin). The significantly larger cell-spreading area observed in cells treated with combined CAP–TMZ (Fig. 6A), results in higher fluorescent pixel intensity and higher αv, αvβ3 and αvβ5 cell surface integrin expression. Thus, the reduced migration rates shown in Fig. 5 for combined CAP–TMZ, correlates positively with increased αv-family surface integrin expression and cell spreading.

## Discussion

Novel cancer treatment strategies aiming to enhance the therapeutic effects of chemotherapy while reducing its dose and side effects are necessary for effectively improving survival outcomes in glioblastoma. Cold atmospheric plasma has consistently exhibited a positive anticancer activity as a stand-alone therapy^7,43,44^ and is being proposed for combination therapy with temozolomide. Major challenges that must be addressed for successful treatment of infiltrative tumors, such as glioblastomas, include: penetration to the tumor site, specificity to cancerous tissue only, and resistance to drug treatment. Conventional therapies are unable to overcome some of these challenges as they primarily rely on vascular delivery of drugs. Several elements, including abnormal vasculature and elevated interstitial fluid pressure, can act to prevent proper drug diffusion leading to poor penetration effect. Unlike drugs, the effect of CAP is not diffusion-dependent because the CAP jet is very focal and can be positioned to treat particular regions in a tumor^30^. The CAP technology has shown specificity towards several types of cancers^45,46^ including glioblastoma^30^. However, the specific parameters for glioblastoma treatment are yet to be elucidated. CAP’s unique composition (ultraviolet rays, electromagnetic fields and RONS)^5,6^ can provide both genotoxic and phototoxic effects. This dual effect can offer a distinctive advantage over radiation therapy which relies primarily on the effect of reactive species. This claim was also supported by a recent study which revealed that plasma could significantly enhance treatment response to radiation therapy^10^.

The present study demonstrates the role of CAP in improving the efficacy of TMZ, the first line chemotherapeutic agent for the treatment of glioblastoma. The effect of CAP therapy has been demonstrated by several basic cellular responses including cell cycle, DNA and mitochondrial damage, protein synthesis, apoptosis, and migration^47,48^. Here, we are presenting glioblastoma cell response to combined CAP–TMZ therapy in the context of alterations in cell viability, cell cycle progression, DNA damage, cell migration and integrin expression. To our knowledge, this is the third study to report on treatment with combined CAP–TMZ^49,50^ and the first investigation on the role of CAP–TMZ in cell surface integrin expression and cell migration in glioblastoma.

According to the results from the cell viability assay, 180 s-CAP treatment successfully restored the effect of the non-cytotoxic TMZ dose (10 μM) and further enhanced the effect of the cytotoxic dose (50 μM) in U87MG cells. Consistent with our previous findings^30^, CAP caused cell cycle interference by inducing a robust increase in the G_2_/M population. However, the role of CAP in intensifying the effect of TMZ was initially unclear, as the percent fraction of cells arrested in G2/M following TMZ and combined CAP–TMZ therapy was similar. The investigation utilizing γ-H2AX (pSer139) revealed that CAP induced a greater degree of DNA damage when combined with TMZ. This expected finding was consistent with prior investigations on the role of CAP in cancer treatment. Earlier studies have reported that treatment with CAP could led to impairment of DNA, cell cycle arrest at G2/M and further induce apoptosis in glioblastoma^30,51^. Attesting to the important role of CAP in improving TMZ efficacy, combined CAP–TMZ treatment caused a more significant inhibition in the rate of cell migration compared to standalone treatments of CAP and TMZ within 24 h of treatment. This observation has been substantiated by previous studies that have reported a decrease in the rate of cell migration after CAP treatment^52,53^. On day 6, cells treated with the combined CAP–TMZ and TMZ displayed similar migration rates likely due to the single CAP treatment having maximized its effect on cell migration over the course of days.

To generate the necessary traction forces required for cell migration, cells depend on adhesive interactions with the ECM substratum, mediated at least in part by integrins^54,55^. Previous studies with CAP on fibroblasts and normal epithelial cells, revealed a significant inhibition of integrin expression that correlated with a decrease in cell migration^19,21,56–58^. Inhibition of cell migration is significant because, in cancer cells, it prevents cancer metastasis and improves survival outcomes^59^. Unlike fibroblasts and epithelial cells, cancer cells are less adhesive and more migratory despite having high cell surface integrin expression. This has been shown to be due to increased cooperation between integrins and growth factor receptors to promote cell proliferation in cancer cells. It can also lead to adherent cells becoming anchorage independent. Cell surface αvβ3 and αvβ5 integrin levels are high in glioma. High integrin expression is associated with poor glioblastoma prognosis and survival. Several in vitro studies have shown that silencing αv integrin expression is associated with increased H2AX phosphorylation and apoptosis^18,60,61^. Silencing cell surface integrins on adherent cells induces a form of apoptosis referred to as anoikis^62^. Cells undergoing anoikis would be expected to sustain more DNA damage in response to treatment compared to cells expressing integrins because they already have initiated apoptosis prior to treatment. Since not all cancer cells retain the ability to undergo anoikis, the integrin silencing studies show that glioma cells retain anchorage dependence.

Our data show CAP–TMZ treated cells have more DNA damage associated with increased cell-spreading and higher αv, αvβ3 and αvβ5 cell surface integrins than CAP or TMZ treated cells. Increased cell surface integrin expression is positively associated with augmented cell adhesion and leads to reduced cell migration^63,64^. Constricted cell migration can impede cancer cell metastasis and proliferation and is positively associated with increased DNA damage^65^. Furthermore, DNA damage may inhibit cell metabolic activity and cause mitochondrial malfunction, eventually leading to apoptosis.

The decrease in cell migration and elevated levels of γ-H2AX observed in CAP–TMZ compared to TMZ treatment alone supports an important role for αv integrins in glioblastoma. CAP–TMZ treatment induces DNA damage while also inducing cells to increase their cell surface integrin expression making cells less metastatic by reducing their ability to migrate. Yet, the increased integrin expression may also promote growth factor mediated cell signaling allowing cells to resist treatment. Further studies aimed at determining how integrin mediated growth factor signaling is impacted by CAP–TMZ are needed to allow us to maximize our ability to treat patients with glioblastoma.

At present, CAP-produced reactive oxygen species (ROS) are one of the main thoroughly investigated chemical components linked to the therapeutic effects of the CAP technology^5,10,23,46,66,67^. However, it remains questionable that plasma-generated ROS can reach the nucleus and damage DNA directly. Hence this concept of the ROS-dependent CAP mechanism of action is starting to shift. Attesting to this observation, it was reported that DNA damage characterized by the phosphorylation of γ-H2AX occurs as a consequence of oxidative stress and apoptosis and it is not caused by the plasma-generated ROS^68^. In a different study conducted with CAP and hydrogen peroxide, it was proposed that CAP creates a state of activation, also referred to as sensitization, for the most part independent of the CAP-produced ROS^69^. Consequently, it is important to recall that CAP can also induce physical effects via ultraviolet rays and electromagnetic fields, both potential mechanisms of action that may better explain the impact of CAP technology in cancer cell death. Attesting to the significant role of CAP emitted electromagnetic waves, is was recently revealed that CAP could cause cell death even when a physical barrier was introduced between the cells and the CAP jet. However, the role of EM waves in CAP–TMZ treatment is yet to be investigated^70^.

Synergism in combination therapy is an additional factor to be considered when investigating the role of CAP therapy in enhancing cell repose to TMZ treatment. A previous study revealed synergism by reporting a more significant cell viability inhibition in combined CAP–TMZ compared to treatment with CAP-only^49^. However, our cell viability data revealed a reverse cell response pattern with CAP leading to a more significant reduction in cell viability than combined CAP–TMZ treatment. Furthermore, contrary to the previous study, our cell cycle data showed no clear difference in the cell distribution at G2/M between TMZ and combined CAP–TMZ treatment. The discrepancies between studies in cell response to CAP–TMZ treatment could be attributed to differences in CAP devices, treatment parameters, and/or assays utilized for testing.

The mechanisms of action of TMZ is an additional significant factor to be taken into account when developing combination therapies with CAP for sensitizing cells to ineffective drug doses. The response to TMZ treatment is often associated with O^6^-methylguanine-DNA methyltransferase (MGMT) expression. MGMT is a repair enzyme that protects cells against DNA damage induced by alkylating agents such as TMZ. Overexpression of the MGMT gene results in resistance to TMZ^71,72^. However, even in the best-case scenarios when the MGMT gene is silent, glioblastoma still remains incurable. Therefore, the application of CAP therapy is meaningful even when tested in MGMT negative cell lines such as the U87MG because it may help improve survival outcomes in patients in the long run. Additional studies are needed to investigate cell response on MGMT positive and negative cell lines and to conduct a more in-depth investigation of the cellular mechanism activated by CAP during TMZ treatment.

Collectively, our data supports the notion that CAP can significantly improve response to TMZ treatment as demonstrated by the reduction in cell viability in the combined CAP–TMZ treated condition. The findings presented in this study introduce CAP as a promising option to be utilized in future investigations for sensitizing cells to TMZ treatment. Ultimately, the question still remains whether combined CAP–TMZ therapy will be effective for patients with glioblastoma. The depth of penetration of the combined CAP–TMZ effect will also have to be assessed while identifying methods for optimizing the physical and chemical properties of plasma to maximize therapeutic efficacy with minimal side-effects.

## Methods

### CAP treatment and device operation parameters

The CAP device was manufactured at the Micro-propulsion and Nanotechnology Laboratory of the George Washington University^73,74^. The device was operated at a frequency of 12.5 kHz with a helium flow rate of 6.5 L per min. The helium plasma jet was generated via streamer propagations along the helium flow field. The spot size of the plasma jet when in contact with liquid media had a diameter of less than 10 mm. The distance between the plasma jet outlet and the surface of the cell culture medium was fixed at 2 cm. The device was operated in the discharge voltage range of 6.2–6.6 kV. Plasma treatment was performed in a direct method with cells in culture media (Fig. 1A).

### Reagents and cell culture

Temozolomide was obtained from Life Sciences (ALX-420-044; Farmingdale, NY). It was resuspended at 20 mg/mL in 100% DMSO, aliquoted and stored at − 20 °C at a concentration of 100 mM. The DMSO concentration in the TMZ, CAP, and combined CAP–TMZ treatment variables was maintained at 0.25% for all experiments. The human cancer cell line, U87MG (glioblastoma) was purchased from the American Type Culture Collection (ATCC) (Manassas, VA). Glioblastoma cells were passaged in cell culture in Dulbecco’s Modified Eagle Medium (DMEM) (Life Technologies) supplemented with 5% (v/v) fetal bovine serum (ThermoFisher) and 1% (v/v) Penicillin–Streptomycin (Life Technologies). Cells were harvested after three passages and were seeded in 24 well plates for performing experiments. The cell culture media utilized for all experiments was prepared with 1% (v/v) fetal bovine serum and 1% (v/v) penicillin–streptomycin.

### CAP and temozolomide treatment

Cells were seeded on 24-well plates at a concentration of 20,000 cells per well and grown overnight with complete DMEM media containing 1% FBS and 1% penicillin–streptomycin for investigating cell response in the presence of TMZ and plasma. After a single plasma treatment of 60 and 180 s, the media was replaced with fresh media and/or TMZ according to testing conditions. Cells were maintained under standard cell culture conditions at 37 °C and 6% CO_2_ (Day 0). The drug and media were replaced on alternate days up to day 6. The following conditions were tested: control (untreated), CAP, TMZ and combined CAP–TMZ (Fig. 2B).

### Cell viability measurement

TMZ concentrations of 10, 25, 50, 100 and 250 μM were evaluated for up to 6 days with the drug being replaced every other day on 24 well plates. TMZ concentrations of 10 and 50 μM were evaluated for additional studies on day 1 and/or day 6 for plasma treatments of 60 and 180 s. The cell viability experiments were preformed three times in six replicates per condition. CellTiter-Glo 2.0 Luminescent Cell Viability Assay (G9242; Promega) was utilized for quantifying the effect of the drug and plasma on cell viability. The assay determines the number of viable cells based on the quantification of ATP, an indicator of metabolically viable cells. The CellTiter-Glo 2.0 reagent was added to each well in equal volumes as that of cell culture media. During the 10-min incubation at room temperature, the reagent led to cell lysis and generation of a luminescent signal proportional to the amount of ATP present. After the 10 min incubation was complete, 200 μL of reagent in media was transferred in triplicate to a 96 well white walled plate and the luminescent signal was recorded with the Tecan microplate reader.

### Flow cytometry and cell cycle analysis

DNA staining was performed for U87MG cells treated for 180 s with plasma and TMZ concentrations of 10 and 50 μM. After 6 days in culture following the procedure described in “CAP and temozolomide treatment”, U87MG cells were harvested and centrifuged at 1200 rpm for 5 min. They were further washed 2× with phosphate-buffered saline (PBS), fixed in ice-cold 70% ethanol and stored at 4 °C for 2–4 days. Cells were washed 2× with PBS and incubated in the dark in PBS containing 20 mg/ml RNase A (Thermo; EN0531) and 50 mg/mL propidium iodide (PI) (Thermo; P3566) at 37 °C for 30 min. Flow cytometry data collection was performed on a BD Celesta Cell Analyzer at the George Washington University’s Flow Cytometry Core Facility. Data analysis for DNA content was performed with FCS 6 Express software (De Novo; Glendale, CA) for quantifying G1, S and G2/M-phases of the cell cycle.

### Cell migration time-lapse studies

Time-lapse studies were performed using an Olympus IX81 microscope (Olympus America, Center Valley, PA) equipped with a motorized stage and a temperature, humidity and CO_2_ controlled chamber. Using relief-contrast optics, images (10×) were obtained every 10 min per well for an uninterrupted 16 h and 40 min (100 images). Images were analyzed with a Metamorph image analysis software (Molecular Devices Corporations, Chicago, IL). Velocities and persistent displacement of ten cells per well that did not undergo mitosis were calculated using the track cell module. A visual basic program assisted in data analysis. An average velocity, net displacement, and total displacement were calculated for each cell tracked. Cell tracking was performed immediately following 180 s of plasma treatment (Day 0–1) and on Day 5–6 with a TMZ concentration of 50 μM.

### αv, αvβ3, and αvβ5 cell surface integrins and γ-H2AX immunofluorescent staining

For experiments involving immunofluorescent staining, cells were plated onto 24 well plates and experiments were performed on day 6. The cell culture media was discarded, and cells were fixed in 4% paraformaldehyde (Thermo; 28906) in PBS for 20 min, and then rinsed in PBS. Non-specific staining of cells was blocked by incubating the cells for 30 min in blocking a buffer (1% Bovine Serum Albumin (BSA), 1% horse serum in 1× PBS). Cells were incubated overnight at 4 °C in the appropriate primary antibodies in the following dilutions: αv; goat derived CD51 antibody (R&D Systems; AF1219, 1:500), αvβ3; mouse derived clone LM609 antibody (EMD Millipore; MAB1976, 1:1000) and αvβ5; mouse derived P1F6 clone antibody (EMD Millipore; MAB1961, 1:2000) followed by 3× 10-min washes with PBS the next day. Additionally, cells were incubated with blocking buffer for 30 min followed by 1-h incubation with their respective secondary antibodies at 1:500 dilution. Nuclei were visualized using DAPI (Invitrogen Corp; D21490) in a 1:2000 dilution. Immunofluorescence detection of γ-H2AX (Ser139) (Cell Signaling; mAb9718) was performed to monitor the formation of DNA double-strand breaks (DSBs). The above protocol was followed and 0.1% Triton-× 100 was added to the blocking buffer. Washing of the cells was performed with PBS-Tween for permeabilizing them. Primary γ-H2AX antibody was added at a 1:200 dilution and the secondary antibody at a 1:500 dilution. Before the addition of DAPI, cells were also stained with Alexa Fluor 594 Phalloidin (ThermoFisher; A12381, 1:5000) for visualizing F-actin. Images were obtained at 20× and 40× magnification using a Nikon Eclipse TS2R and sum pixel intensity for each image was quantified using NIS Elements BR v5.00 software. (Melville, NY).

### Imaging and statistical analysis

Data plots and statistical analyses were performed with Prism 8 (GraphPad software, San Diego, CA, USA). The differences between groups were determined by ANOVA (Analysis of Variance) and data were considered significant for p values of less than 0.05. When standard deviations between groups were not equal, significance was determined using Welch corrected unpaired t-tests. Potential outlier values for all conditions were determined with an online outlier calculator from GraphPad which performs the Grubb's test (ESD method). Statistical significance was determined as: ***(p-value < 0.001), **(p-value < 0.01) and *(p-value < 0.05). Adobe Photoshop was used to manage the images for all figures.

## Conclusion

In conclusion, the cytotoxicity of TMZ was amplified in the presence of CAP as indicated by the cell viability data. Combined CAP–TMZ treatment induced G2/M cell cycle arrest and increased DNA damage measured by γ-H2AX. The addition of CAP to TMZ treatment also caused an inhibition in cell migration and increased αvβ3 and αvβ5 cell surface integrin expression. The findings reveal compelling evidence in support of the important role of CAP in mediating cancer cell suppression in the presence of temozolomide.

## References

[1] Friedman HS, Kerby T, Calvert H (2000). Temozolomide and treatment of malignant glioma. Clin. Cancer Res..

[2] Lee SY (2016). Temozolomide resistance in glioblastoma multiforme. Genes Dis..

[3] Haar CP (2012). Drug resistance in glioblastoma: A mini review. Neurochem. Res..

[4] Jiapaer S, Furuta T, Tanaka S, Kitabayashi T, Nakada M (2018). Potential strategies overcoming the temozolomide resistance for glioblastoma. Neurol. Med. Chir..

[5] Keidar M (2013). Cold atmospheric plasma in cancer therapy. Phys. Plasmas.

[6] Dubuc, A.*et al.* Use of cold-atmospheric plasma in oncology: A concise systematic review. *Therap. Adv. Med. Oncol.* **10**, 1758835918786475 (2018). 10.1177/1758835918786475 PMC6055243 30046358

[7] Chen Z (2017). A novel micro cold atmospheric plasma device for glioblastoma both in vitro and in vivo. Cancers (Basel)..

[8] Partecke LI (2012). Tissue Tolerable Plasma (TTP) induces apoptosis in pancreatic cancer cells in vitro and in vivo. BMC Cancer.

[9] Lin AG (2018). Non-thermal plasma induces immunogenic cell death in vivo in murine CT26 colorectal tumors. Oncoimmunology.

[10] Lin L (2018). Non-thermal plasma inhibits tumor growth and proliferation and enhances the sensitivity to radiation in vitro and in vivo. Oncol. Rep..

[11] Hou, J. *et al.* Non-thermal plasma treatment altered gene expression profiling in non-small-cell lung cancer A549 cells. *BMC Genom.* **16**(1), 435 (2015). 10.1186/s12864-015-1644-8 PMC4483225 26116417

[12] von Woedtke T, Reuter S, Masur K, Weltmann K-D (2013). Plasmas for medicine. Phys. Rep..

[13] Metelmann HR (2018). Clinical experience with cold plasma in the treatment of locally advanced head and neck cancer. Clin. Plasma Med..

[14] Kalghatgi S, Fridman A, Azizkhan-Clifford J, Friedman G (2012). Damage in mammalian cells by non-thermal atmospheric pressure microsecond pulsed dielectric barrier discharge plasma is not mediated by ozone. Plasma Process. Polym..

[15] Kim C-H (2010). Induction of cell growth arrest by atmospheric non-thermal plasma in colorectal cancer cells. J. Biotechnol..

[16] Aoudjit F, Vuori K (2012). Integrin signaling in cancer cell survival and chemoresistance. Chemother. Res. Pract..

[17] Malric L (2017). Interest of integrins targeting in glioblastoma according to tumor heterogeneity and cancer stem cell paradigm: an update. Oncotarget.

[18] Christmann M (2017). Integrin αVβ3 silencing sensitizes malignant glioma cells to temozolomide by suppression of homologous recombination repair. Oncotarget.

[19] Volotskova O, Stepp MA, Keidar M (2012). Integrin activation by a cold atmospheric plasma jet. New J. Phys..

[20] Volotskova O, Shashurin A, Stepp MA, Pal-Ghosh S, Keidar M (2011). Plasma-controlled cell migration: Localization of cold plasma-cell interaction region. Plasma Med..

[21] Shashurin A (2010). Influence of cold plasma atmospheric jet on surface integrin expression of living cells. Plasma Process. Polym..

[22] Vandamme M (2011). Response of human glioma U87 xenografted on mice to non thermal plasma treatment. Plasma Med..

[23] Vandamme M (2012). ROS implication in a new antitumor strategy based on non-thermal plasma. Int. J. Cancer.

[24] Li Y (2017). Effects of atmospheric-pressure non-thermal bio-compatible plasma and plasma activated nitric oxide water on cervical cancer cells. Sci. Rep..

[25] Liedtke KR (2017). Non-thermal plasma-treated solution demonstrates antitumor activity against pancreatic cancer cells in vitro and in vivo. Sci. Rep..

[26] Nakamura K (2017). Novel intraperitoneal treatment with non-thermal plasma-activated medium inhibits metastatic potential of ovarian cancer cells. Sci. Rep..

[27] Liu Y (2017). Selective effects of non-thermal atmospheric plasma on triple-negative breast normal and carcinoma cells through different cell signaling pathways. Sci. Rep..

[28] Yu H (2018). Paclitaxel-loaded core-shell magnetic nanoparticles and cold atmospheric plasma inhibit non-small cell lung cancer growth. ACS Appl. Mater. Interfaces.

[29] Cheng X (2014). The effect of tuning cold plasma composition on glioblastoma cell viability. PLoS ONE.

[30] Siu A (2015). Differential effects of cold atmospheric plasma in the treatment of malignant glioma. PLoS ONE.

[31] Patel M, McCully C, Godwin K, Balis FM (2003). Plasma and cerebrospinal fluid pharmacokinetics of intravenous temozolomide in non-human primates. J. Neurooncol..

[32] Castro GN (2015). Effects of temozolomide (TMZ) on the expression and interaction of heat shock proteins (HSPs) and DNA repair proteins in human malignant glioma cells. Cell Stress Chaperones.

[33] Ostermann S (2004). Plasma and cerebrospinal fluid population pharmacokinetics of temozolomide in malignant glioma patients. Clin. Cancer Res..

[34] Anderson, D. *et al.*. Comparison of two methods for measuring γ-H2AX nuclear fluorescence as a marker of DNA damage in cultured human cells: Applications for microbeam radiation therapy. *J. Instrum.* **8**(06), C06008 (2013).

[35] Armento, A., Ehlers, J., Schötterl, S. & Naumann, U. Molecular mechanisms of glioma cell motility. in *Glioblastoma* 73–93 (Codon Publications, 2017). 10.15586/codon.glioblastoma.2017.ch5 29251855

[36] Demuth T, Berens ME (2004). Molecular mechanisms of glioma cell migration and invasion. J. Neurooncol..

[37] Hynes RO (2002). Integrins: Bidirectional, allosteric signaling machines. Cell.

[38] Martin S, Janouskova H, Dontenwill M (2012). Integrins and p53 pathways in glioblastoma resistance to temozolomide. Front. Oncol..

[39] Thomas GJ (2001). Expression of the αvβ6 integrin promotes migration and invasion in squamous carcinoma cells. J. Invest. Dermatol..

[40] Paolillo M, Serra M, Schinelli S (2016). Integrins in glioblastoma: Still an attractive target?. Pharmacol. Res..

[41] Hodkinson PS (2006). ECM overrides DNA damage-induced cell cycle arrest and apoptosis in small-cell lung cancer cells through β1 integrin-dependent activation of PI3-kinase. Cell Death Differ..

[42] Park J (2016). Non-thermal atmospheric pressure plasma efficiently promotes the proliferation of adipose tissue-derived stem cells by activating NO-response pathways. Sci. Rep..

[43] Chen, Z. *et al.*. Cold atmospheric plasma discharged in water and its potential use in cancer therapy. *J. Phys. D. Appl. Phys.* **50**(1), 015208 (2017).

[44] Ratovitski EA (2014). Anti-cancer therapies of 21st century: Novel approach to treat human cancers using cold atmospheric plasma. Plasma Process. Polym..

[45] Iseki S (2012). Selective killing of ovarian cancer cells through induction of apoptosis by nonequilibrium atmospheric pressure plasma. Appl. Phys. Lett..

[46] Keidar M (2011). Cold plasma selectivity and the possibility of a paradigm shift in cancer therapy. Br. J. Cancer.

[47] Keidar M, Yan D, Beilis II, Trink B, Sherman JH (2018). Plasmas for treating cancer: Opportunities for adaptive and self-adaptive approaches. Trends Biotechnol..

[48] Gjika E (2018). Adaptation of operational parameters of cold atmospheric plasma for in vitro treatment of cancer cells. ACS Appl. Mater. Interfaces.

[49] Köritzer J (2013). Restoration of sensitivity in chemo—Resistant glioma cells by cold atmospheric plasma. PLoS ONE.

[50] Conway GE (2016). Non-thermal atmospheric plasma induces ROS-independent cell death in U373MG glioma cells and augments the cytotoxicity of temozolomide. Br. J. Cancer.

[51] Akter M, Jangra A, Choi SA, Choi EH, Han I (2020). Non-thermal atmospheric pressure bio-compatible plasma stimulates apoptosis via p38/MAPK mechanism in U87 malignant glioblastoma. Cancers (Basel)..

[52] Privat-Maldonado, A., Gorbanev, Y., Dewilde, S., Smits, E. & Bogaerts, A. Reduction of human glioblastoma spheroids using cold atmospheric plasma: The combined effect of short-and long-lived reactive species. *Cancers (Basel).* **10**, (2018). 10.3390/cancers10110394 PMC6266784 30360539

[53] Wang M (2013). Cold atmospheric plasma for selectively ablating metastatic breast cancer cells. PLoS ONE.

[54] Hood JD, Cheresh DA (2002). Role of integrins in cell invasion and migration. Nat. Rev. Cancer.

[55] Bello L (2003). IS20I, a specific αvβ3 integrin inhibitor, reduces glioma growth in vivo. Neurosurgery.

[56] Blackert S, Haertel B, Wende K, von Woedtke T, Lindequist U (2013). Influence of non-thermal atmospheric pressure plasma on cellular structures and processes in human keratinocytes (HaCaT). J. Dermatol. Sci..

[57] Haertel B (2012). Surface molecules on HaCaT keratinocytes after interaction with non-thermal atmospheric pressure plasma. Cell Biol. Int..

[58] Naciri M, Dowling D, Al-Rubeai M (2014). Differential sensitivity of mammalian cell lines to non-thermal atmospheric plasma. Plasma Process. Polym..

[59] Lefranc F, Brotchi J, Kiss R (2005). Possible future issues in the treatment of glioblastomas: Special emphasis on cell migration and the resistance of migrating glioblastoma cells to apoptosis. J. Clin. Oncol..

[60] Meldolesi J (2016). Pharmacology of the cell/matrix form of adhesion. Pharmacol. Res..

[61] Bianconi D (2016). Integrins in the spotlight of cancer. Int. J. Mol. Sci..

[62] Frisch SM, Ruoslahti E (1997). Integrins and anoikis. Curr. Opin. Cell Biol..

[63] Huttenlocher A, Horwitz AR (2011). Integrins in cell migration. Cold Spring Harb. Perspect. Biol..

[64] Mei CM, Shen S (2009). The roles of cell adhesion molecules in tumor suppression and cell migration: A new paradox. Cell Adhesion Migration.

[65] Pfeifer CR (2018). Constricted migration increases DNA damage and independently represses cell cycle. Mol. Biol. Cell.

[66] Bauer, G., Sersenová, D., Graves, D. B. & Machala, Z. Cold atmospheric plasma and plasma-activated medium trigger RONS-based tumor cell apoptosis. *Sci. Rep.* **9**(1), 1–28 (2019). 10.1038/s41598-019-50291-0 PMC6775051 31578342

[67] Keidar, M. Plasma for cancer treatment. *Plasma Sources Sci. Technol.* **24**, (2015).

[68] Bekeschus, S. *et al.* Elevated H2AX phosphorylation observed with kINPen plasma treatment is not caused by ROS-mediated DNA damage but is the consequence of apoptosis. *Oxid. Med. Cell. Longev.* (2019). 10.1155/2019/8535163 PMC6770374 31641425

[69] Yan, D. *et al.* The cell activation phenomena in the cold atmospheric plasma cancer treatment. *Sci. Rep.* **8**(1), 1–10 (2018). 10.1038/s41598-018-33914-w PMC6194007 30337623

[70] Yan, D. *et al.* A physically triggered cell death via transbarrier cold atmospheric plasma cancer treatment. *ACS Appl. Mater. Interfaces* **12**(31), 34548–34563 (2020). 10.1021/acsami.0c06500 32648738

[71] Barciszewska A-M, Gurda D, Głodowicz P, Nowak S, Naskręt-Barciszewska MZ (2015). A new epigenetic mechanism of temozolomide action in glioma cells. PLoS ONE.

[72] Zhang, J., F.G. Stevens, M. & D. Bradshaw, T. Temozolomide: Mechanisms of action, repair and resistance. *Curr. Mol. pharmacol.* **5**(1), 102–114 (2012). 10.2174/1874467211205010102 22122467

[73] Lin L, Lyu Y, Trink B, Canady J, Keidar M (2019). Cold atmospheric helium plasma jet in humid air environment. J. Appl. Phys..

[74] Lin L, Lyu Y, Shneider MN, Keidar M (2018). Average electron temperature estimation of streamer discharge in ambient air. Rev. Sci. Instrum..

